# Non-participation in breast cancer screening for women with chronic diseases and multimorbidity: a population-based cohort study

**DOI:** 10.1186/s12885-015-1829-1

**Published:** 2015-10-26

**Authors:** L. F. Jensen, A. F. Pedersen, B. Andersen, M. Vestergaard, P. Vedsted

**Affiliations:** Department of Public Health, The Research Unit for General Practice, Aarhus University, Bartholins Allé 2, 8000 Aarhus C, Denmark; Department of Public Health, Section for General Practice, Aarhus University, Bartholins Allé 2, 8000 Aarhus C, Denmark; Department of Public Health, The Research Centre for Cancer Diagnosis in Primary Care (CaP), Aarhus University, Bartholins Allé 2, 8000 Aarhus C, Denmark; Department for Public Health Programs, Regional Hospital of Randers, Skovlyvej 1, 8930 Randers, Denmark

**Keywords:** Chronic disease, Multimorbidity, Breast cancer screening, Mammography screening, Participation, Non-attendance, Denmark

## Abstract

**Background:**

Chronic diseases and multimorbidity are common in western countries and associated with increased breast cancer mortality. This study aims to investigate non-participation in breast cancer screening among women with chronic diseases and multimorbidity and the role of time in this association.

**Method:**

This population-based cohort study used regional and national registries. Women who were invited to the first breast cancer screening round in the Central Denmark Region in 2008–09 were included (*n* = 149,234). Selected chronic diseases and multimorbidity were assessed up to 10 years before the screening date. Prevalence ratios (PR) were used as an association measure.

**Results:**

The results indicated that women with at least one chronic condition were significantly more likely not to participate in breast cancer screening. In adjusted analysis, a significantly higher likelihood of non-participation was found for women with cancer (PR = 1.50, 95 % CI: 1.40–1.60), mental illness (PR = 1.51, 95 % CI: 1.42–1.60), chronic obstructive pulmonary disease (PR = 1.51, 95 % CI: 1.42–1.62), neurological disorders (PR = 1.24, 95 % CI: 1.12–1.37) and kidney disease (PR = 1.70, 95 % CI 1.49–1.94), whereas women with chronic bowel disease (PR = 0.75, 95 % CI 0.65–0.88) were more likely to participate than women without these disease. Multimorbidity was associated with increased non-participation likelihood. E.g. having 3 or more diseases was associated with 58 % increased non-participation likelihood (95 % CI: 27–96 %). Higher non-participation was also observed for women with severe multimorbidity (PR = 1.53, 95 % CI: 1.23–1.90) and mental-physical multimorbidity (PR = 1.54, 95 % CI: 1.36–1.75).

**Conclusion:**

In conclusion, we found a strong association between non-participation in breast cancer screening for some chronic diseases and for multimorbidity. The highest propensity not to participate was observed for women with hospital contacts related to the chronic disease in the period closest to the screening date.

**Electronic supplementary material:**

The online version of this article (doi:10.1186/s12885-015-1829-1) contains supplementary material, which is available to authorized users.

## Background

Breast cancer is the second most common cancer type worldwide and the most common cancer type among Danish women [1, 2]. Breast cancer screening can detect breast cancer at an early stage where the prognosis for survival is better [3]. Breast cancer screening has therefore been introduced as a universal programme in many western countries. In Denmark, women between 50 and 69 years of age are invited biennially to a mammogram free of charge [3].

A growing proportion of people are living with chronic diseases and multimorbidity [4, 5]. Studies have found that comorbidity increases the mortality risk among breast cancer patients [6, 7] which in some studies have been related to the comorbidities rather than to the breast cancer [6, 8]. Yet, the cancer prognosis depends on the disease stage at the time of diagnosis [3], and given the increased mortality rate among breast cancer patients with chronic diseases, this group may benefit particularly from early diagnosis.

The association between chronic diseases, multimorbidity and non-participation in breast cancer screening has not been studied sufficiently [9, 10]. Some studies have investigated diseases individually and their results are not consistent [9–14]. Five studies found that multimorbidity increased non-participation [9, 13, 15–17], but Heflin *et al.* [10] found in their study that three or more conditions increased the propensity to participate.

Although chronic diseases are long-lasting by definition, patients may experience periods where the disease is not followed at hospital but rather by primary care, e.g., during a stable disease period [18]. We hypothesised that being diagnosed with diseases that involve hospital contact close to the screening date affects screening behaviour more than the presence of chronic diseases without recent hospital contact. To our knowledge, this issue has not been studied before.

This study has two purposes: first, to analyse whether being diagnosed with specific chronic diseases or with multimorbidity is associated with non-participation in breast cancer screening and, second, to study whether any such association varies with respect to the time that has elapsed since the disease required contact to the hospital sector with the investigated diseases. We hypothesised that women with chronic diseases and multimorbidity were more likely not to participation.

## Methods

### Setting

The setting for the study was the Central Denmark Region (1.2 million inhabitants, approx. 150,000 women aged 50–69). Breast cancer screening was introduced as an organised, universal and free-of-charge programme in 2008–09 in the Central Denmark Region where 78.9 % of the invited women participated [19].

### Study design and population

We conducted an observational, registry-based, historical cohort study with screening participation as the outcome and we assessed registrations of chronic diseases up to ten years before the scheduled screening date. The population comprised women invited to the first organised breast cancer screening round in the Central Denmark Region in 2008–09 (*N* = 149,234) and we excluded women who were dead or have moved between the invitations were send out and the screening date or were outside the caption area (*n* = 324) and women with registration of breast cancer in the Danish Cancer Registry [20] (*n* = 4,646). In total, 144,264 women were included; see more information in our previous publication [19].

### Data collection and variables

Information on participation in breast cancer screening was obtained from a regional administrative database containing individual information on, e.g., participation status, the scheduled screening date and the unique central registration number (CRN) possessed by all Danes [21]. The present study is based on data from the prevalent screening round in the Central Denmark Region. Hence, a woman was defined as a participant if she had participated in the first organised breast cancer screening round in the Central Denmark Region and as a non-participant if she had not.

All data described in this section were linked using the unique CRN number [21].

Data on chronic diseases were drawn from the Danish National Patient Registry (NPR) [22]. The registry was founded in 1977 and initially included admission information. Since 1995, the registry has expanded to include information on all outpatient and emergency contacts. All contacts are registered with a main diagnosis (i.e. action diagnosis) based on the International Classification of Diseases, 10th version (ICD-10) [22]. Data on psychiatric diseases were drawn from the Danish Psychiatric Central Research Register (PCRR). All Danish psychiatric departments document every contact to the PCRR, and ICD-10 codes for each hospital admission, outpatient and emergency contacts were available for the entire study period [23].

The chronic diseases of interest were selected based on a recent literature review [24], which recommended the inclusion of 11 core chronic diseases when assessing multimorbidity. Based on their recommendations and another study in the field [25], we included a larger number of specific chronic diseases and grouped these diseases into 11 comprehensive chronic disease groups (CDGs) on which data were drawn from the NPR and the PCRR. The following CDGs were included: diabetes, hypertension, cancer, chronic obstructive pulmonary disease (COPD), cardiovascular diseases, chronic arthritis, chronic kidney disease, chronic liver disease, chronic neurological disorders, chronic bowel disease and chronic mental illness (Additional file 1).

Multimorbidity was operationalised as follows: “Multimorbidity” covers the co-occurrence of two or more chronic diseases from two or more of the CDGs. “Severe multimorbidity” designates the co-occurrence of three or more chronic diseases from three or more of the CDGs. “Physical multimorbidity” describes the co-occurrence of two or more physical CDGs, but without the mental CDG. “Physical-mental multimorbidity” signifies the co-occurrence of at least one physical CDG and the mental CDG. Thus, a woman could be categorised as having more than one type of multimorbidity; e.g. severe multimorbidity and physical multimorbidity. Finally, we measured “disease counts” within the categories: 0, 1, 2, ≥3 CDGs, with the latter category being combined due to few occurrences.

Study participants were categorised with one of the diseases mentioned above if they had an emergency contact, an outpatient contact or an admission with one of the selected diseases to any Danish hospital during the 10 years period before screening. We intended to study if an association varied with time as we hypothesised that the likelihood of non-participation would be stronger for women with a chronic disease requiring hospital attention in the period leading up to screening compared to women with chronic diseases that did not require hospital attention in the period leading up to screening. Because of this, data on the 10-year follow-up were stratified into two time periods which were not mutually exclusive: (1) any hospital contact with the included chronic diseases ≤2 years (i.e. 0–730 days) before the screening date; and (2) hospital contacts with the included chronic diseases >2–10 years (i.e. 731–3652 days) before the screening date. Thus, a woman could be categorised in both groups if she was registered in the NPR or PCRR with a given disease in both time periods. E.g.55 % of all women was registered with rheumatoid arthritis in both time periods (data not shown).

We obtained individual data on the population’s socio-economic position (SEP) registered the year of the scheduled screening date from Statistics Denmark [26] and included: *ethnicity* categorised as 1) Danish and descendants of immigrants and 2) immigrants. *Marital status* was categorised as married/cohabitating and single. *Education* was classified according to the UNESCO classification [27] as low (≤10 years), middle (11–15 years) and higher (>15 years). Finally, *age* on the date of the scheduled screening was included as a continuous variable in the multivariate analyses.

Finally, almost all Danish citizens (98 %) are listed with a specific general practitioner (GP) or general practice [28], and data on GP attachment were obtained from the Danish National Health Service Registry which was used to do cluster adjustments by GP affiliation.

### Statistical analysis

All analyses were performed using Stata 13.1. Prevalence ratios (PRs) with 95 % confidence intervals (95 % CI) were estimated using generalised linear models (GLM) [29, 30]. PRs were chosen over the odds ratio, as it has been found to overestimate associations when the outcome is frequent [30].

Unadjusted analyses were conducted for each of the CDGs. We compared women having each specific CDG with women without the CDG in question. In model 1, we adjusted for SEP (ethnicity, marital status, education and age). In model 2, we adjusted for the variables in model 1 (SEP) and for the coexistence of the other included diseases. We also hypothesised that an association between one given disease and non-participation in one time period could be confounded by having chronic diseases in the other time period. Therefore, we also adjusted model 2 for being registered in the NPR or PCRR in the other time period.

Unadjusted analyses were also conducted for the multimorbidity variables. Model 1 adjusted for SEP (ethnicity, marital status, education and age). Model 2 adjusted for the variables in model 1 (SEP) and for being registered in the NPR or PCRR with multimorbidity in the other time period. E.g. when studying severe multimorbidity >2–10 years before the scheduled screening date, we adjusted for SEP and for having severe multimorbidity in the period ≤2 years before the scheduled screening date.

We assessed the association between the latest hospital contact with either of the included diseases and non-participation with a cubic spline model, using the method proposed by Orsini and Greenland and knots were set at 5, 27.5, 50, 72.5 and 95 percentiles [31, 32].

All analyses were assessed with robust variance estimates to adjust for clustering of patients in general practices. This was done as practice clustering might be related to the propensity to be diagnosed with a chronic disease and also to participate in breast cancer screening [33].

### Ethical approvals

No ethical approval was required according to Danish legislation and the National Committee on Health Research Ethics in the Central Denmark Region as the study was based on registry and survey data (j. no. 181/2011). Approval for data on screening behaviour was granted from the Central Denmark Regions legal department (j. no.: 1-16-02-109-09) and permission for the national registry data was granted from by the Danish Data Protection Agency (j. no.: 2009-41-3471).

## Results

### Study population social-characteristics

A higher non-participation proportion was found among women in the oldest age group, single women, women with non-Danish origin and with low education (Table 1).

**Table 1:** Socio-economic position of the study population divided according to participation in the screening programme (*n* = 144,264, numbers vary due to missing observations)

All women	Participants		Non-participants		P-value (chi^2^)
	N (% column)		N (% column)		
	113,811	(79.1)	30,453	(21.1)	
Chronic disease (no missings)^a^					<0.001
No	104,012	(79.7)	26,533	(20.3)	
Yes	9,799	(71.4)	3,920	(28.6)	
Age on the screening date (no missings)					<0.001
50–54 years	30,965	(80.4)	7,536	(19.6)	
55–59 years	30,722	(80.2)	7,580	(19.8)	
60–64 years	30,532	(79.2)	7,998	(20.8)	
65–69 years	21,592	(74.6)	7,339	(25.4)	
Marital status (missing *N* = 83 (0.06 %))					<0.001
Married/cohabiting	88,590	(82.7)	18,484	(17.3)	
Single	25,183	(67.9)	11,924	(32.1)	
Ethnicity (missing *N* = 48 (0.03 %))					<0.001
Danish/descendant	110,018	(79.6)	28,201	(18.3)	
Immigrants	3,773	(62.9)	2,224	(37.1)	
Education (missing *N* = 2,279 (1.6 %))					<0.001
≤ 10 years	39,214	(75.6)	12,651	(24.4)	
11–15 years	47,661	(81.8)	10,624	(18.2)	
> 15 years	25,549	(80.2)	6,286	(19.7)	

(*n* = 144,264, numbers vary due to missing observations)

^a^Presence of chronic diseases ≤2 years before the scheduled screening date

### CDGs and non-participation in breast cancer screening

In total, 20.3 % of women without a chronic disease did not participate in the first screening round whereas 28.6 % women with minimum one of the chronic diseases did not participate (Table 1). For most of the CDGs, women who had a chronic condition were more inclined to abstain from participation than women who had no chronic diseases except for hypertension and chronic arthritis. The participation proportion was higher for women with chronic bowel disease than for women without chronic bowel disease (Table 2).

**Table 2:** Distribution of women with the selected CDGs ≤2 years and >2–10 years before screening and screening participation (*n* = 144,264)

	Contacts to hospital with the CDGs ≤2 years before the screening date					Contacts to hospital with the CDGs >2–10 years before the screening date				
Chronic diseases	Participants		Non-participants			Participants		Non-participants		
	N	(% row)	N	(% row)	P-value (chi^2^)	N	(% row)	N	(% row)	P-value (chi^2^)
Cardiovascular diseases					<0.001					<0.001
No	111,578	(79.0)	29,670	(21.0)		108,916	(79.0)	28,885	(21.0)	
Yes	2,233	(74.0)	783	(26.0)		4,895	(75.7)	1,568	(24.3)	
Cancer					<0.001					<0.001
No	112,517	(79.1)	29,780	(20.9)		111,848	(79.0)	29,798	(21.0)	
Yes	1,294	(65.8)	673	(34.2)		1,963	(75.0)	655	(25.0)	
Hypertension					0.944					0.844
No	112,475	(78.9)	30,097	(21.1)		110,753	(78.9)	29,641	(21.1)	
Yes	1,336	(78.9)	356	(21,0)		3,058	(79.0)	812	(21.0)	
Chronic mental illness					<0.001					<0.001
No	112,683	(79.1)	29,696	(20.9)		111,261	(79.3)	28,970	(20.7)	
Yes	1,128	(59.8)	757	(40.2)		2,550	(63.2)	1,483	(36.8)	
Diabetes					<0.001					<0.001
No	112,116	(79.0)	29,836	(21.0)		111,801	(79.1)	29,609	(20.9)	
Yes	1,695	(73.3)	617	(36.7)		2,010	(70.4)	844	(29.6)	
Chronic obstructive pulmonary disease					<0.001					<0.001
No	112,975	(79.1)	29,917	(20.9)		112,439	(79.1)	29,709	(20.9)	
Yes	836	(60.9)	536	(39.1)		1,372	(64.8)	744	(35.2)	
Chronic neurological disorders					<0.001					<0.001
No	113,234	(78.9)	30,188	(21.1)		113,049	(79.0)	30,078	(21.0)	
Yes	577	(68.5)	265	(31.5)		762	(67.0)	375	(33.0)	
Chronic arthritis					0.079					0.439
No	113,083	(78.9)	30,254	(21.1)		112,865	(78.9)	30,186	(21.1)	
Yes	728	(78.5)	199	(21.5)		946	(78.0)	267	(22.0)	
Chronic bowel disease					0.004					<0.001
No	113,185	(78.9)	30,326	(21.1)		112,905	(78.9)	30,272	(21.1)	
Yes	626	(83.1)	127	(16.9)		906	(83.3)	181	(16.7)	
Chronic liver disease					<0.001					<0.001
No	113,606	(78.9)	30,367	(21.1)		113,567	(78.9)	30,343	(21.1)	
Yes	205	(70.4)	86	(29.6)		244	(68.9)	110	(31.1)	
Chronic kidney disease					<0.001					<0.001
No	113,684	(78.9)	30,341	(21.1)		113,655	(78.9)	30,355	(21.1)	
Yes	127	(53.1)	112	(46.9)		156	(61.4)	98	(38.6)	

In model 2, having cancer, mental illness, COPD, chronic neurological disorder and chronic kidney disease significantly increased the likelihood of non-participation in both time periods, whereas having bowel disease increased the likelihood of participation (PR_model2_ = 0.75, 95 % CI: 0.65–0.88) (Table 3). Diabetes was insignificantly related to screening ≤2 years before the screening date; but if the latest hospital contact was between >2–10 years before the screening date, women with diabetes were significantly less likely to participate. For women with chronic liver disease and cardiovascular disease, non-participation became insignificant in model 2. Hypertension and chronic arthritis were significantly associated with higher participation if the last hospital contact was >2–10 years before the screening date, but not if it was ≤2 years before screening (Table 3).

**Table 3:** PR of screening non-participation by the selected CDGs ≤2 years and >2–10 years before screening (*n* = 144,264)

	Unadjusted		Model 1^a^		Model 2^b^	
	≤2 years	>2–10 years	≤2 years	>2–10 years	≤2 years	>2–10 years
	PR (95 % CI)	PR (95 % CI)	PR (95 % CI)	PR (95 % CI)	PR (95 % CI)	PR (95 % CI)
Cardiovascular diseases						
No	1 (ref)	1 (ref)	1 (ref)	1 (ref)	1 (ref)	1 (ref)
Yes	**1.24 (1.16–1.31)**	**1.16 (1.11–1.21)**	**1.13 (1.06–1.21)**	1.04 (0.99–1.09)	1.05 (0.99–1.13)	0.98 (0.93–1.02)
Cancer						
No	1 (ref)	1 (ref)	1 (ref)	1 (ref)	1 (ref)	1 (ref)
Yes	**1.63 (1.54–1.74)**	**1.19 (1.11–1.27)**	**1.56 (1.46–1.66)**	**1.16 (1.08–1.24)**	**1.50 (1.40–1.60)**	**1.10 (1.03–1.17)**
Hypertension						
No	1 (ref)	1 (ref)	1 (ref)	1 (ref)	1 (ref)	1 (ref)
Yes	1.00 (0.91–1.09)	0.99 (0.93–1.06)	0.97 (0.87–1.07)	0.97 (0.91–1.04)	0.92 (0.83–1.02)	**0.93 (0.87–0.99)**
Chronic mental illness						
No	1 (ref)	1 (ref)	1 (ref)	1 (ref)	1 (ref)	1 (ref)
Yes	**1.93 (1.82–2.04)**	**1.78 (1.71–1.86)**	**1.62 (1.53–1.71)**	**1.51 (1.44–1.57)**	**1.51 (1.42–1.60)**	**1.40 (1.35–1.46)**
Diabetes						
No	1 (ref)	1 (ref)	1 (ref)	1 (ref)	1 (ref)	1 (ref)
Yes	**1.27 (1.19–1.36)**	**1.41 (1.33–1.50)**	**1.14 (1.06–1.22)**	**1.23 (1.17–1.31)**	1.05 (0.97–1.13)	**1.10 (1.04–1.17)**
Chronic obstructive pulmonary disease						
No	1 (ref**)**	1 (ref)	1 (ref)	1 (ref)	1 (ref)	1 (ref)
Yes	**1.87 (1.75–1.99)**	**1.68 (1.59–1.78)**	**1.62 (1.52–1.73**)	**1.43 (1.35–1.52)**	**1.51 (1.42–1.62)**	**1.32 (1.24–1.40)**
Chronic neurological disorders						
No	1 (ref)	1 (ref)	1 (ref)	1 (ref)	1 (ref)	1 (ref)
Yes	**1.50 (1.35–1.65)**	**1.57 (1.44–1.71)**	**1.36 (1.23–1.50)**	**1.40 (1.29–1.52)**	**1.24 (1.12–1.37)**	**1.23 (1.13–1.34)**
Chronic arthritis						
No	1 (ref)	1 (ref)	1 (ref)	1 (ref)	1 (ref)	1 (ref)
Yes	1.02 (0.90–1.15)	1.04 (0.94–1.16)	0.97 (0.86–1.10)	0.99 (0.89–1.11)	0.90 (0.80–1.02)	**0.89 (0.80–1.00)**
Chronic bowel disease						
No	1 (ref)	1 (ref)	1 (ref)	1 (ref)	1 (ref)	1 (ref)
Yes	**0.80 (0.68–0.94)**	**0.79 (0.69–0.90)**	**0.81 (0.69–0.94)**	**0.78 (0.68–0.89)**	**0.75 (0.65–0.88)**	**0.71 (0.61–0.81)**
Chronic liver disease						
No	1 (ref)	1 (ref)	1 (ref)	**1 (ref)**	1 (ref)	1 (ref)
Yes	**1.40 (1.17–1.67)**	**1.47 (1.26–1.72)**	1.20 (1.00–1.43)	**1.24 (1.06–1.43)**	1.12 (0.93–1.34)	1.08 (0.92–1.26)
Chronic kidney disease						
No	1 (ref)	(ref)	1 (ref)	1 (ref)	1 (ref)	1 (ref)
Yes	**2.22 (1.94–2.55)**	**1.83 (1.57–2.14)**	**1.93 (1.69–2.20)**	**1.61 (1.36–1.90)**	**1.70 (1.49–1.94)**	**1.40 (1.19–1.65)**

^a^Adjusted for age, ethnicity, marital status and education

^b^Adjusted for age, ethnicity, marital status, education, for being registered in the NPR or PCRR with the other studied chronic diseases, and for being registered in the NPR or PCRR with one of the CDGs in the other time period (yes/no). E.g. cancer 0–2 year before screening is adjusted for SEP, for the remaining 10 studied diseases and for being in hospital contact with one of the 11 included chronic disease groups >2–10 years before screening

Statistically significant results in bold

### Multimorbidity and non-participation in breast cancer screening

Overall, non-participation was more common among women with multimorbidity and a higher disease count (Table 4). In the regression analyses, the disease-count variable showed an increased non-participation likelihood with each additional disease compared with no disease (e.g. 3 diseases: PR_model 2_ = 1.58, 95 % CI: 1.27–1.96) (Table 5). Women with any of the multimorbidity aspects were significantly more likely not to participate than were women without multimorbidity. This applied in both time periods, but the estimates were highest for hospital contact ≤2 years before the screening date. In general, the associations between the different types of multimorbidity and non-participation were largely similar. However, having severe multimorbidity (≥3 diseases) was associated with a somewhat higher non-participation propensity (PR_model2_ = 1.53, 95 % CI: 1.23–1.90) than having multimorbidity (≥2 diseases) (PR_model2_ = 1.38, 95 % CI: 1.29–1.49). Non-participation likelihood was also somewhat higher for women with physical-mental multimorbidity than women with physical multimorbidity (PR_model2_ = 1.54, 95 % CI: 1.36–1.75 and PR_model2_ = 1.37, 95 % CI: 1.26–1.49, respectively) (Table 5).

**Table 4:** Distribution of women with multimorbidity ≤2 years and >2–10 years before screening and their participation status (*n* = 144,264)

	Contacts to hospital with the CDGs ≤2 years before the screening date					Contacts to hospital with the CDGs >2–10 years before the screening date				
	Participants		Non-participants			Participants		Non-participants		
	N	(% row)	N	(% row)	P-value (chi^2^)	N	(% row)	N	(% row)	P-value (chi^2^)
Disease count					<0.001					<0.001
0	104,012	(79.7)	26,533	(20.3)		97,440	(79.8)	24,598	(20.2)	
1	8,887	(72.4)	3,383	(27.6)		14,153	(74.8)	4,763	(25.2)	
2	845	(63.4)	488	(36.6)		1,966	(67.7)	939	(32.3)	
≥ 3	67	(57.8)	49	(42.2)		252	(62.2)	153	(37.8)	
Multimorbidity (≥2 diseases)					<0.001					<0.001
No	112,899	(79.0)	29,916	(21.0)		111,593	(79.2)	29,361	(20.8)	
Yes	912	(62.9)	537	(37.1)		2,218	(67.0)	1,092	(33.0)	
Severe multimorbidity (≥3 diseases)					<0.001					<0.001
No	113,744	(78.9)	30,404	(21.1)		113,559	(78.9)	30,300	(21.1)	
Yes	67	(57.8)	49	(42.2)		252	(62.2)	153	(37.8)	
Physical multimorbidity					<0.001					<0.001
No	113,043	(79.0)	30,032	(21.0)		112,045	(79.0)	29,654	(20.9)	
Yes	768	(64.6)	421	(35.4)		1,766	(68.8)	799	(31.2)	
Physical-mental multimorbidity					<0.001					<0.001
No	113,657	(78.9)	30,320	(21.1)		113,269	(79.0)	30,099	(21.0)	
Yes	154	(53.7)	133	(46.3)		542	(60.5)	354	(39.5)

**Table 5:** PR of screening non-participation by multimorbidity ≤2 years and >2–10 years before screening (*n* = 144,264)

	Unadjusted		Model 1^a^		Model 2^b^	
	≤2 years	>2–10 years	≤2 years	>2–10 years	≤2 years	>2–10 years
	PR (95 % CI)	PR (95 % CI)	PR (95 % CI)	PR (95 % CI)	PR (95 % CI)	PR (95 % CI)
Disease count						
0	1 (ref)	1 (ref)	1 (ref)	1 (ref)	1 (ref)	1 (ref)
1	**1.36 (1.32–1.40)**	**1.25 (1.22–1.28)**	**1.25 (1.21–1.29)**	**1.14 (1.11–1.18)**	**1.20 (1.16–1.24)**	**1.08 (1.04–1.11)**
2	**1.80 (1.68–1.39)**	**1.60 (1.52–1.69)**	**1.55 (1.45–1.67)**	**1.38 (1.31–1.45)**	**1.47 (1.36–1.58)**	**1.25 (1.18–1.32)**
≥ 3	**2.08 (1.68–2.57)**	**1.87 (1.65–2.12)**	**1.69 (1.36–2.09)**	**1.52 (1.33–1.73)**	**1.58 (1.27–1.96)**	**1.33 (1.17–1.52)**
Multimorbidity (≥2 diseases)						
No	1 (ref)	1 (ref)	1 (ref)	1 (ref)	1 (ref)	1 (ref)
Yes	**1.77 (1.65–1.89)**	**1.58 (1.51–1.66)**	**1.52 (1.42–1.63)**	**1.36 (1.30–1.43)**	**1.38 (1.29–1.49)**	**1.28 (1.22–1.35)**
Severe multimorbidity (≥3 diseases)						
No	1 (ref)	1 (ref)	1 (ref)	1 (ref)	1 (ref)	1 (ref)
Yes	**2.00 (1.62–2.48)**	**1.79 (1.58–2.03)**	**1.63 (1.31–2.02)**	**1.47 (1.29–1.67)**	**1.53 (1.23–1.90)**	**1.44 (1.26–1.64)**
Physical multimorbidity						
No	1 (ref)	1 (ref)	1 (ref)	1 (ref)	1 (ref)	1 (ref)
Yes	**1.69 (1.56–1.82)**	**1.49 (1.40–1.58)**	**1.47 (1.36–1.59)**	**1.30 (1.23–1.38)**	**1.37 (1.26–1.49)**	**1.23 (1.16–1.31)**
Physical-mental multimorbidity						
No	1 (ref)	1 (ref)	1 (ref)	1 (ref)	1 (ref)	1 (ref)
Yes	**2.20 (1.94–2.49)**	**1.88 (1.73–2.04)**	**1.80 (1.60–2.02)**	**1.52 (1.40–1.66)**	**1.54 (1.36–1.75)**	**1.42 (1.30–1.56)**

^a^Adjusted for age, ethnicity, marital status and education

^b^Adjusted for age, ethnicity, marital status, education, and for being registered in the NPR or PCRR with the studied multimorbidity in the other time period (yes/no). E.g. severe multimorbidity 0–2 year is adjusted for SEP, and for being in hospital contact with 3 or more chronic disease groups >2-10 years before screening

Statistically significant results in bold

The association between the latest hospital contacts with any of the included diseases indicated a non-linear association. Non-participation was highest when the latest hospital occurred in the year leading up to screening and the PR of non-participation did not differ markedly if the latest hospital contact occurred 6 or more years before screening (data not shown).

## Discussion

This large population-based cohort study revealed that women with cancer, mental illness, COPD, neurological disorders or kidney disease had an increased likelihood of non-participation in breast cancer screening. The likelihood of non-participation increased with the number of co-existing diseases and was particularly high for women with severe multimorbidity and mental-physical multimorbidity. The associations were in general strongest when the women had hospital based contacts with the disease in the period ≤2 years before screening compared to when the contacts had occurred > 2 to 10 years before screening. Sub-analysis indicated that the likelihood of non-participation was affected the most if the latest hospital contact occurred up to one year before screening.

### Strengths and limitations

Data on screening participation were obtained from an administrative registry with no missing information and no reliance on self-reported data. The cohort comprised a well-defined population, i.e. the prevalent screening round in the Central Denmark Region; and using the CRN, we were able to identify and follow the entire population and can therefore exclude selection bias.

We included chronic diseases as registered in nationwide registries containing information on all hospital-related contacts in Denmark [22, 23]. The validity of the registries has been established for several of the included diseases [34–36]. However, these registers do not contain information on treatment in general practice; thus the results of this study apply only to patients with hospital-requiring treatment. Patients who are treated only in general practice will in our analysis appear in the reference group but because our reference group is very large this proportion will presumably be small and will probably not affect the results markedly. As no Danish registers contains data on routine treatment from general practice, it was not possible to study or adjust for this in the present study.

In this study, chronic diseases and multimorbidity were specified based on leading studies in the field [24, 25]. However, generalisations cannot be made about chronic diseases in general as the study included only some and not all existing chronic diseases. The chronic diseases that were selected were categorised into groups of chronic diseases (see Additional file 1) since we believe that the practical implications of the results would be relevant to more people. Moreover, combining, e.g., several mental diseases with a varying degree of severity and chronicity might lead to less nuanced findings for this group of patients.

### Chronic diseases, multimorbidity and screening behaviour

A previous cancer diagnosis (other than breast cancer) was related to non-participation in both time periods. Several explanations for this finding can be suggested. First of all, some women may have attended a post-cancer follow-up programme and therefore did not find it relevant to participate in screening. Others may have been too ill to participate, or they may have been in a palliative phase. Some may have been unrealistically optimistic [37] and may not have perceived themselves as being at risk of being diagnosed with yet another type of cancer. Conversely, others may have avoided anything relating to cancer because of the trauma experienced by having the first cancer diagnosis. Notwithstanding, it has been shown that some previous cancer types increases the risk of developing later breast cancer [38–40], which makes these results important as this group may benefit particularly from early detection of cancer.

Having chronic bowel disease was the only CDG that significantly increased the propensity to participate in breast cancer screening in both time periods. Chronic bowel disease often has an early onset in life compared with the other included diseases and being diagnosed with this disease often involves continuous health-care follow-up. These women may therefore have been ‘schooled’ from early on in life to engage in healthy lifestyles and may be used to having various contacts with the healthcare system and undergoing tests [41, 42]. A study from 2014 also found higher likelihood of having followed a recommended breast cancer screening programme among women with digestive disease compared to women without any digestive disease [13]. Taken together, this seems to indicate that having a bowel/digestive-related disease has a positive impact on screening behaviour. Having cardiovascular diseases was not associated with non-participation after adjustments, a finding which is supported by other studies [11, 43] but not all [13]. Having hypertension, chronic arthritis and diabetes up to 2 years before screening were not associated with screening participation. However, if the latest contact was >2–10 years before screening, having hypertension and arthritis increased participation significantly, and having diabetes increased non-participation. The underlying mechanisms here are unclear, but they could, e.g., be related to these women’s lifestyles, an issue which should be studied further.

This study shows that having multimorbidity increases non-participation. This is supported by five other studies [9, 13, 15–17], even if one of these studies found an association for women ≥75 years only [17]. Another study, conducted in the USA and based on self-reported data, found the opposite; namely that multimorbidity increased participation [10]. The authors argue that women with multimorbidity have more frequent contact with the GP who plays a direct role in advising women to participate in screening in the USA [10]. However, the other studies which found the opposite were also conducted in the USA.

The association between chronic diseases, multimorbidity and non-participation may be explained by several factors. Women with one or more chronic diseases may not feel well enough to participate, and the competing demands in relation to managing their chronic disease(s) may take up all their energy [9, 18]. It has also been raised as a possible explanation that non-participation among diseased women is based on deliberate decisions from the patient (or their GP) based on an evaluation of costs and benefits, taking into account the severity of the disease, reduced quality of life and shortened life expectancy [9, 15].

This study is the first to include different time periods for hospital contacts. For most diseases and all aspects of multimorbidity, the estimates were strongest for the ≤ 2 year period before the screening date. Some studies have adjusted for e.g. “number of years with illness/in contact with clinic” or “number of contacts” [9, 13, 15, 16], but no study has evaluated whether the associations depend on elapsing time since last hospital contact. Thus, these results add to the current literature as it highlights that non-participation is especially challenged when the woman is affected by the disease in the period leading up to the screening appointment.

This study clearly indicates that women with some of the studied chronic diseases and women with multimorbidity are not engaging in screening to the same extent as their female counterparts who do not have the selected CDGs and multimorbidity. As these women are more frequently in contact with the health care system, this may make it easier to inform them about the advantages of screening for early diagnosis. This group of women may also derive particular benefits from their general practitioner playing a more active role discussing with them the pros and cons of breast cancer screening.

## Conclusion

In conclusion, this study indicates increased non-participation in breast cancer screening for women who previously have been in contact with the secondary healthcare system for some of the selected CDGs. Non-participation was strongly associated with having multimorbidity. Strongest associations were found when the hospital contacts for the diseases had occurred in the recent period before screening. Women suffering from chronic diseases or multimorbidity may achieve health gains from early detection of cancer owing to their participation in screening; and their participation could with possible benefit be encouraged by healthcare professionals.

## Additional file

Additional file 1:
**Selected chronic disease groups and the included diseases in each group together with their ICD-10 codes [** 24 **,** 25 **,** 37 **,** 44 **–** 47 **].** (DOCX 18 kb)

## Abbreviations

CDG: Chronic disease group
CI: Confidence interval
COPD: Chronic Obstructive Pulmonary Disease
CRN: Civil registration number
GLM: Generalised linear models
GP: General practitioner
ICD-10: The International Classification of Diseases, 10th version
NPR: National Patient Registry
PCRR: Danish Psychiatric Central Research Register
PR: Prevalence ratio
SEP: Socio-economic position
