# Supplementary material for: Non-participation in breast cancer screening for women with chronic diseases and multimorbidity: a population-based cohort study
Source: BMC Cancer. 2015 Oct 26;15:798. doi: 10.1186/s12885-015-1829-1 (PMC4623919; doi:10.1186/s12885-015-1829-1)
Supplement: Additional file 1: — Selected chronic disease groups and the included diseases in each group together with their ICD-10 codes [ 24 , 25 , 37 , 44 – 47 ]. (DOCX 18 kb) [file 12885_2015_1829_MOESM1_ESM.docx]

**Additional file 1**. Selected chronic disease groups and the included diseases in each group together with their ICD-10 codes

| **Included CDGs (grey) and included diseases (white)** | **ICD-10 codes and references to included codes** | **Included in Diederichs *et al* (24) or Barnetts *et al* (25)** | |
| --- | --- | --- | --- |
| **Cardiovascular diseases** | (44) | | (24, 25) |
| Ischaemic heart disease  Apoplexy  Acute myocardial infarction  Angina pectoris  Heart failure  Cardiac valve diseases  Atrial fibrillation | I24-I25  I60-I69, G45, G46  I21,I22  I20  I50, I11.0, I13.0, I13.2  I08, I09, I38, I39, Z95  I48 | |  |
| **Cancer** | (45) | | (24, 25) |
| Cancer excluding breast cancer and  malignant skin neoplasms | C00-C97 (ex. C50 and C44) | |  |
| **Hypertension** | (45, 46) | | (24, 25) |
| Hypertension | I10-I15 | |  |
| **Chronic mental illness** | (44, 45) | | (24, 25) |
| Schizophrenia | F20 | | (25) |
| Psychotic disorders | F22-F25, F28-F29 | | (25) |
| Affective disorders incl. depression | F30-39 | | (25) |
| Dementia | F00-F03, F05.1, G30 | | (24, 25) |
| Anxiety  Eating disorder | F40-F41  F50.0, F50.2 | | (25)  (25) |
| **Diabetes** | (45, 46) | | (24, 25) |
| Diabetes | E10-E14 | |  |
| **Chronic obstructive pulmonary disease** | (47) | | (24, 25) |
| COPD | DJ40, DJ409, DJ41, DJ410, DJ411, DJ418, DJ42, DJ429, DJ429A, DJ429B, DJ43, DJ430, DJ430A, DJ431, DJ431A, DJ432, DJ438, DJ439, DJ439A, DJ44, DJ440, DJ441, DJ448, DJ448A, DJ448B, DJ449, DJ47, DJ479, DJ96, DJ960, DJ961, DJ969 | |  |
| **Chronic neurological disorders** | (45, 46) | | (25) |
| Epilepsy  Parkinson’s disease  Multiple sclerosis | G40 (ex. G40.4), G41  G20-G22  G35 | | (25)  (25) |
| **Chronic arthritis** | (46) | | (24, 25) |
| Rheumatoid arthritis | M05, M06, M790 | |  |
| **Inflammatory bowel disease/Chronic bowel disease** | | | (25) |
| Colitis ulcerosa  Mb. Crohn | K51  K50 | |  |
| **Chronic liver disease** | (46) | | (25) |
| Chronic viral hepatitis  Chronic liver disease | B18  K70, K71.3-K71.5, K71.7, K72.1, K72.7, K72.9, K73-K74, K76 | | (25)  (25) |
| **Chronic kidney disease** |  | | (25) |
| Chronic kidney disease | N18+N19 | |  |
